# Functional neuroanatomy of developmental dyslexia: the role of orthographic depth

**DOI:** 10.3389/fnhum.2014.00347

**Published:** 2014-05-20

**Authors:** Fabio Richlan

**Affiliations:** Centre for Neurocognitive Research and Department of Psychology, University of SalzburgSalzburg, Austria

**Keywords:** brain, developmental dyslexia, fMRI, language, neuroimaging, orthography, PET, reading

## Abstract

Orthographic depth (OD) (i.e., the complexity, consistency, or transparency of grapheme-phoneme correspondences in written alphabetic language) plays an important role in the acquisition of reading skills. Correspondingly, developmental dyslexia is characterized by different behavioral manifestations across languages varying in OD. This review focuses on the question of whether these different behavioral manifestations are associated with different functional neuroanatomical manifestations. It provides a review and critique of cross-linguistic brain imaging studies of developmental dyslexia. In addition, it includes an analysis of state-of-the-art functional neuroanatomical models of developmental dyslexia together with orthography-specific predictions derived from these models. These predictions should be tested in future brain imaging studies of typical and atypical reading in order to refine the current neurobiological understanding of developmental dyslexia, especially with respect to orthography-specific and universal aspects.

In this Review Article I will discuss current advances and future directions in the neurobiological understanding of developmental dyslexia. For this purpose, I will focus on brain imaging studies and will elaborate on the question of whether different behavioral manifestations of dyslexia across languages may be associated with different functional neuroanatomical manifestations. This question was not dealt with in previous review articles in the field (e.g., Pugh et al., 2000; Temple, 2002; McCandliss and Noble, 2003; Démonet et al., 2004; Heim and Keil, 2004; Sandak et al., 2004; Shaywitz and Shaywitz, 2005; Schlaggar and McCandliss, 2007). Of main interest will be whether orthographic depth (OD)—a well-known factor in reading acquisition—has an influence on the brain activation pattern during non-impaired and dyslexic reading. For this purpose, I will begin with a review of relevant studies, followed by a critique of some of these studies. Although the focus of the present paper is on OD in alphabetic writing systems, I will also cover cross-cultural studies comparing alphabetic with syllabic or logographic writing systems. This topic is of immediate interest as it can contribute to the understanding of universal and orthography-specific neurobiological manifestations of developmental dyslexia (Frost, 2012). Finally, I will put forward some model-based orthography-specific predictions of classical as well as newer functional neuroanatomical conceptions of developmental dyslexia, which may serve as blueprint for future hypothesis-driven brain imaging studies.

## Orthographic depth and reading acquisition in alphabetic writing systems

OD refers to the complexity, consistency, or transparency of grapheme-phoneme correspondences in written alphabetic language (Frost et al., 1987). A deep (or highly complex or inconsistent or opaque) orthography like English is characterized by multi-letter graphemes, context-dependent rules, and morphological effects resulting in a many-to-many mapping of graphemes to phonemes. In contrast, a shallow (or little complex or consistent or transparent) orthography like Finnish is characterized by consistent one-to-one mapping of graphemes to phonemes (Seymour et al., 2003).

OD has been identified as one of the most important environmental factors influencing learning to read (e.g., Seymour et al., 2003; Landerl et al., 2013). It has a direct effect on how easy or difficult it is for children to translate a new letter string into a phonological code by which phonological word forms can be accessed. The idea is that in deep orthographies phonology has to be retrieved from stored memory representations (i.e., from an internal lexicon), whereas in shallow orthographies phonology can be derived relatively easily and directly from print. The ability to translate letter strings into a phonological code is called phonological recoding and was labeled the *sine qua non* of reading acquisition. It provides the prerequisite for a self-teaching mechanism that enables a young reader to autonomously establish an orthographic lexicon (Share, 1995).

It has been shown numerous times that children exhibit different behavioral performance according to the language they are learning to read. The usual finding is a marked word and pseudoword reading accuracy advantage of children learning to read in a shallow orthography (e.g., Dutch, Finnish, German, Greek, Italian, Spanish) over children learning to read in a deep orthography (English). This pertains to non-impaired (e.g., Wimmer and Goswami, 1994; Cossu et al., 1995; Frith et al., 1998; Aro and Wimmer, 2003; Seymour et al., 2003; Bergmann and Wimmer, 2008; Zoccolotti et al., 2009; Georgiou et al., 2012) as well as to impaired reading acquisition (i.e., developmental dyslexia) (e.g., Wimmer, 1993; Landerl et al., 1997; Landerl and Wimmer, 2000; Spinelli et al., 2005; Zoccolotti et al., 2005; Barca et al., 2006; Davies et al., 2007; Wimmer and Schurz, 2010).

Some accounts, however, emphasize the commonalities between reading in deep and shallow orthographies over their differences (e.g., Ziegler et al., 2003, 2010; Caravolas et al., 2012, 2013). For example, Ziegler et al. (2010) investigated whether the role of different cognitive predictors of reading development (phonological awareness (PA), rapid automatized naming (RAN), phonological short-term memory, vocabulary, and nonverbal IQ) varies with OD. They showed that, although its influence is weaker in shallow compared with deep orthographies, PA is a relatively universal predictor of reading performance in alphabetic languages. Likewise, developmental dyslexia was characterized by similar deficits (overall slow reading, increased difficulties with nonwords compared with words, and slow and effortful phonological decoding) in a shallow orthography (German) and a deep orthography (English) (Ziegler et al., 2003).

With respect to the developmental pattern of cognitive predictors, Vaessen et al. (2010) found a strong contribution of PA to reading fluency in beginning readers, followed by a gradual shift towards stronger contribution of RAN in more proficient readers. Importantly, this general developmental shift was not influenced by OD of the three studied languages (Hungarian, Dutch, Portuguese). The contribution of PA to reading fluency, however, was important for a longer period of time in deeper orthographies. Likewise, Moll et al. (2014) confirmed that PA and RAN both account for significant amounts of unique variance in literacy development across five orthographies (English, French, German, Hungarian, Finnish). In all studied languages, PA was the best predictor of reading accuracy and spelling, whereas RAN was the best predictor of reading speed. With respect to developmental dyslexia, Landerl et al. (2013) showed that both PA and RAN were strong concurrent predictors of reading problems. The influence of PA and RAN was larger in deeper orthographies, in which more participants were correctly classified as dyslexic. In sum, the results suggest that the same cognitive components underlie reading development in deep and shallow orthographies, but to a different degree that varies as a function of reading level.

One attempt to explain the differences in reading speed and reading accuracy across orthographies is psycholinguistic grain size theory (Ziegler and Goswami, 2005, 2006). This theory postulates that the behavioral differences can be attributed to differences in the size of the orthographic units on which phonological recoding is based. Specifically, readers in shallow orthographies can rely on small psycholinguistic grain size (i.e., single letters or letter clusters corresponding to single phonemes) because grapheme-phoneme correspondences are relatively consistent. In contrast, readers of deep orthographies additionally have to rely on larger psycholinguistic grain size (i.e., letter patterns corresponding to rimes, syllables, or even whole words), which are more consistent compared with the relatively inconsistent grapheme-phoneme correspondences. It was shown that especially the continuous switching between small unit recoding and large unit recoding strategies leads to the reading accuracy disadvantage in deep orthographies (Goswami et al., 2003). In addition, it is far more difficult for a beginning reader to remember the mapping from orthography to phonology based on the vast amount of letter pattern-rime/syllable correspondences compared with the limited number of grapheme-phoneme correspondences.

## Orthographic depth and brain imaging in alphabetic writing systems

Cross-linguistic brain imaging studies are extremely laborious and difficult to conduct. They require well-matched designs and samples and face many practical problems (e.g., availability and comparability of assessment tools, differences in the school system, socio-economic factors, matching of stimuli, data acquisition protocols, etc.). Therefore, it is not surprising that to date only few cross-linguistic brain imaging studies have been published. Another approach focuses on bilingual or biliterate participants and searches for the effect of OD on brain activation within participants. The findings from these two types of studies will be reviewed below. In addition, there are promising attempts to investigate the influence of OD by means of artificial language training studies (Mei et al., 2013; Taylor et al., 2014). These studies can potentially contribute to the understanding of language-related differences in reading development.

Motivated by findings on behavioral differences between readers in deep and shallow orthographies, Paulesu et al. (2000) conducted a seminal positron emission tomography (PET) study. They compared brain activation during word and nonword reading in Italian and English skilled adult readers. With a conjunction analysis, they identified a largely left-lateralized brain network showing common activation in both groups. Specifically, this network included left inferior frontal (IFG), precentral (PreG), fusiform (FFG), inferior (ITG) and middle temporal (MTG) regions as well as bilateral superior temporal gyri (STG).

In addition, orthography-specific effects were investigated in a direct comparison between Italian and English readers. In general, orthography-specific effects were reflected in quantitative rather than qualitative differences in brain activation. That is, the very same brain regions were active in both languages, but to a different degree and spatial extent. Specifically, the direct comparison identified the left posterior STG at the junction to the parietal cortex with higher activation in Italian readers and the left posterior ITG and anterior IFG with higher activation in English readers. The STG activation was interpreted as reflecting enhanced involvement of phonological processing, whereas the ITG and IFG activation was interpreted as reflecting enhanced involvement of the orthographic lexicon. The left IFG region was also associated with semantic processing. In sum, the results were taken as evidence for the shaping of the neurobiological systems for reading through specific properties (i.e., OD) of the written language.

In a follow-up study, Paulesu et al. (2001) investigated whether such language-related brain activation effects would also pertain to developmental dyslexia. They acquired PET scans from non-impaired and dyslexic university students from Italy, France, and the UK during the same activation tasks used in their earlier study. The main finding was the identification of a large left hemisphere cluster comprising STG, MTG, and ITG as well as middle occipital gyri with higher activation in non-impaired readers compared with dyslexic readers, irrespective of orthography. Vice versa, no regions were identified with higher activation in dyslexic readers compared with non-impaired readers.

With respect to orthography-specific effects, Paulesu et al. (2001) could confirm their earlier findings on non-impaired reading. That is, they replicated the findings on the English non-impaired readers with the French non-impaired readers (which they classified as readers in a deep orthography). Crucially, however, no orthography-specific effects were found in the direct comparison of the dyslexic subsamples from the three languages varying in OD. Taken together with the fact that all of the dyslexic participants were selected based on a marked phonological deficit, the brain imaging results were interpreted as evidence for a universal neurocognitive basis of developmental dyslexia.

Subsequently, Silani et al. (2005) acquired structural magnetic resonance imaging (MRI) data in addition to the functional PET activation data of the Italian, French, and English participants of Paulesu et al. (2001). Their voxel-based morphometry (VBM) analysis sought to investigate the correspondence between regional dysfunctions (i.e., underactivation) and anatomical alterations (i.e., with respect to the cortical surface and underlying fibers) in developmental dyslexia. For gray matter density, consistent reduction in dyslexic readers across all three orthographies was identified in the left MTG (with consistent augmentation in an adjacent left posterior MTG region). For white matter density, consistent reduction in dyslexic readers across all three orthographies was identified underneath the left IFG, postcentral, and supramarginal cortices. Similar to the functional activation study, no orthography-specific effects of dyslexia were found.

A different approach to investigate orthography-specific effects in reading-related brain activation was recently put forward by Das et al. (2011). They studied mono- and biliterate English and Hindi adult readers during reading aloud English and Hindi words. Furthermore, the group of biliterate readers were divided into those who learnt to read both languages at the age of 5 (simultaneous biliterate readers) and those who learnt to read Hindi at the age of 5 and English at the age of 10 (sequential biliterate readers). Crucially, only biliterate adults who learnt to read both languages simultaneously at the age of 5 showed a similar activation pattern as monoliterates, that is, left ITG activation for English (deep orthography) and left inferior parietal lobule (IPL) activation for Hindi (shallow orthography). During English word reading, the sequential biliterates did not exhibit the left ITG activation found in simultaneous biliterates and English monoliterates. The divergence between simultaneous and sequential biliterate readers speaks for early orthography-specific functional tuning of reading networks in the brain that persists into adulthood. This unique fMRI study is particularly impressive because it shows orthography-specific effects within participants rather than between participants.

Similarly, Bar-Kochva and Breznitz (2012) used a within-subjects design to investigate the effect OD on brain activation during reading by means of event-related potentials (ERPs). They studied Hebrew speakers, which were familiar with two forms of script (pointed and unpointed) varying in OD. During a lexical decision task, the shallow pointed script evoked larger amplitudes around 165 ms over occipito-temporal (OT) electrodes, whereas the deep unpointed script evoked larger amplitudes around 340 ms over occipito-parietal electrodes. The same authors found these effects also in adult dyslexic readers, but with reduced and delayed amplitudes. These results were interpreted as a failure in dyslexic readers to fine-tune their reading strategies to the particular demands imposed by the deep and shallow orthographies (Bar-Kochva and Breznitz, 2014).

## Brain imaging comparing alphabetic with syllabic or logographic writing systems

In addition to comparing written alphabetic languages with varying OD, there were attempts to compare brain activation of proficient readers in alphabetic writing systems with Chinese (logographic), Japanese Kana (syllabic), and Japanese Kanji (morpho-syllabic). In their coordinate-based meta-analysis, Bolger et al. (2005) found convergence of reading-related activation of all four writing systems in left STG, IFG, and OT regions. As expected, the activation patterns of the different writing systems also differed to some degree, mainly with respect to extension of clusters. Specifically, divergence was identified in a posterior aspect of the left STG (with higher activation for Western and Kana writing systems), in an anterior aspect of the left IFG (with higher activation for Chinese), and in the right OT cortex (again with higher activation for Chinese). The higher activation for the alphabetic and syllabic writing systems in the left posterior STG was interpreted as reflecting the mapping of written symbols to fine-grained speech sounds (phonemes and syllables)—in contrast to mapping to whole-word phonology in the case of Chinese and Kanji. The higher activation for Chinese (logographic) in the left anterior IFG was interpreted as reflecting synchronous processing of semantic and phonological information that is—due to the high number of homophones in Chinese—required for unambiguous identification of written symbols. Finally, the higher activation for Chinese in the right OT cortex was interpreted as reflecting global and low spatial frequency processing of the spatial arrangement of the written symbols. In line with these meta-analytic findings, a newer near-infrared spectroscopy (NIRS) study comparing English and Chinese readers during a homophone judgment task identified higher activation in the left STG in English readers and higher activation in the left middle frontal gyrus (MFG) in Chinese readers (Chen et al., 2008).

Another study with biliterate participants in Korean Hangul (phonographic) and Hanja (logographic) showed that the level of reading proficiency in the logographic orthography modulated the reading strategy and, correspondingly, the brain activation pattern during processing of the phonographic orthography (Jeon, 2012). Specifically, highly skilled readers, relying on the lexical route, activated anterior cingulate, MFG, and OT regions, whereas less skilled readers, relying on the sublexical route, activated IPL and IFG regions.

A developmental difference between English and Chinese readers was recently observed in an fMRI study using a word pair rhyme judgment task (Brennan et al., 2013). A network of left hemisphere regions (including STG, IPL, and IFG) showed an increase of activation in English adults compared with children but not in Chinese adults compared with children. This finding was taken as evidence for reorganization of the left hemisphere phonological network in readers of alphabetic but not in readers of logographic writing systems, possibly as a result from the differences in psycholinguistic grain size, with smaller units in English compared with Chinese.

First evidence for writing system-related brain activation abnormalities in developmental dyslexia was reported by Siok et al. (2004). They found marked underactivation of the left MFG in dyslexic compared with non-impaired Chinese children during homophone judgment and lexical decision. Further indication for a crucial influence of the writing system on the neurobiological manifestation of developmental dyslexia was provided in a follow-up study (Siok et al., 2008). Chinese dyslexic children not only exhibited functional underactivation of the left MFG in response to a rhyme judgment task but also showed reduced gray matter volume of this region compared with their age-matched non-impaired peers. Interestingly, the Chinese dyslexic readers did not exhibit the left posterior underactivation, which is distinctive of dyslexic readers in alphabetic writing systems. The unique engagement of the left MFG in Chinese non-impaired reading (and failure of engagement in dyslexia) was explained by the strong involvement of motor processes during learning to read Chinese. Children in primary school spend a lot of time copying newly learned characters and this likely involves recruitment of the left MFG just anterior to the motor cortex. It was shown that handwriting skills are the best predictor of reading ability, with both supported by long-term graphomotor memories of characters (Tan et al., 2005).

Although these data seem to challenge the universality of neurocognitive explanations of dyslexia, Ziegler (2006) argues that the phonological deficit theory still accounts for the problems of Chinese dyslexic readers. Instead of the mapping of graphemes onto phonemes, the phonological deficit of Chinese dyslexics lies in the association of complex graphomotor programs of logographs to whole-word phonology. That is, the universality of the phonological deficit is still valid, but its putative association with a left STG dysfunction is not. Another possibility is that the left MFG—as part of the central executive—subserves a coordination and integration function of orthographic, phonological, and semantic information, which is particularly important for Chinese reading (Perfetti et al., 2006).

In a direct cross-linguistic comparison, it was recently shown that the brain activation differences between dyslexic and non-impaired readers of Chinese and English are not that massive as previously thought. In a well-conceived fMRI study, Hu et al. (2010) found writing system-specific activation differences during a semantic word matching task between the two groups of non-impaired readers but not between the two groups of dyslexic readers. Specifically, Chinese non-impaired readers exhibited higher activation compared with English non-impaired readers in the left IFG sulcus and lower activation in left posterior superior temporal sulcus. Crucially, dyslexic readers of both languages showed reliable activation in these two regions, indicating the use of a similar reading strategy. The dyslexic readers shared, however, a common pattern of underactivation relative to non-impaired readers in the left MFG, left posterior MTG, left angular gyrus, and left OT sulcus. Thus, the functional neuroanatomical manifestation of dyslexia in English and Chinese is similar when a reading task with demands on semantic processing is used.

## Methodological considerations

Cross-linguistic brain imaging studies of developmental dyslexia have also been the target of serious criticism. For example, Hadzibeganovic et al. (2010) questioned the biological unity account of dyslexia because of both conceptual and methodological issues of some of the above mentioned studies. In particular, they focused their critique on the seminal studies by Paulesu et al. (2001) and Silani et al. (2005), which had the biggest impact on the field. The problems raised include (i) missing subtyping of dyslexia cases; (ii) differences in selection of participants across the three countries; and (iii) discounting of differences in cognitive demands for reading diverse orthographies.

Some of the points of criticism of Hadzibeganovic et al. (2010) are valid; however, I want to clarify the crucial aspects by providing some explanations for why the studies of the Paulesu group did not identify orthography-specific effects in the neurobiology of developmental dyslexia. First of all—and most importantly—Paulesu et al. (2001) and Silani et al. (2005) did not claim that all developmental dyslexics have the same brain abnormality. Rather, they showed that there is some shared component across the three alphabetic orthographies (Italian, French, English)—namely left posterior underactivation as well as reduced gray and white matter density. Although their finding of an absence of orthography-specific effects is suggestive of a complete overlap of brain abnormality patterns, the much more probable scenario—based on evidence from studies comparing different writing systems (e.g., Bolger et al., 2005; Hu et al., 2010)—is that there is some core dysfunction present in dyslexia in all writing systems with additional language-related variations and extensions. This means that there is a shared component (the core dysfunction), but with orthography-specific differences based on the particular properties of the language and the reader’s experience. It is plausible to assume that the nature of orthography-specific differences is quantitatively rather than qualitatively. That is, that differential weighing of cognitive components is reflected mainly in the degree and spatial extent of activation clusters rather than in variation of anatomical location (Paulesu et al., 2000). There are several reasonable explanations for why the Paulesu et al. (2001) and Silani et al. (2005) studies did not identify these fine-grained language-related variations. These possibilities will be spelled out in detail below.

**Figure 1:**
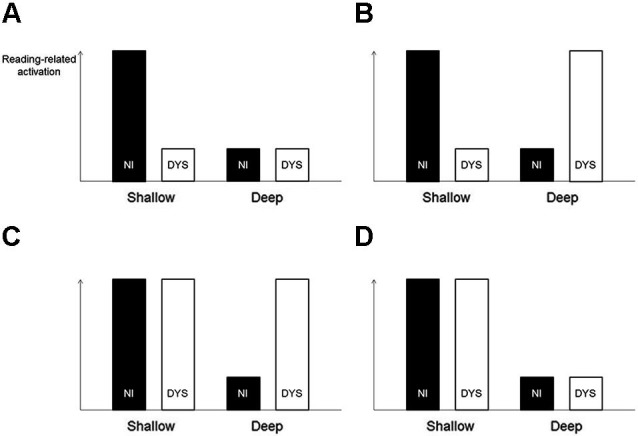
**Illustration of different activation patterns of non-impaired (NI) and dyslexic (DYS) readers**. The strategy to compare abnormality patterns of dyslexic readers (relative to non-impaired readers) across languages would correctly identify the situations illustrated in sections **(A–C)** as showing an orthography-specific abnormality pattern and would correctly reject the situation illustrated in section **(D)**. In contrast, the actual strategy used by Paulesu et al. (2001) (i.e., comparing dyslexic activation directly across languages), would only correctly identify the situation illustrated in section **(B)**. It would fail to identify the situations illustrated in sections **(A)** and **(C)** and would incorrectly identify the situation illustrated in section **(D)**.

The logic behind Paulesu et al.’s search for orthography-specific effects in developmental dyslexia was not ideal. Paulesu et al. (2001) directly compared the activation patterns of the dyslexic readers across the three languages. It would be, however, more sensible to compare the abnormality patterns of the dyslexic readers (relative to the non-impaired readers) across the three languages. As an example, imagine the following situation (illustrated in Figure 1A): Italian but not English non-impaired readers show strong activation of the left STG. Both Italian and English dyslexic readers show weak activation of the left STG. In the case of the Italian dyslexics, let us assume that this weak activation is the result of a specific deficit of a cognitive process supported by this region, whereas in the case of the English dyslexics, it is simply the result of little requirement by the deep orthography of English to engage this very same process (hence only weak activation in non-impaired readers as well). A direct comparison of Italian versus English dyslexic readers—the strategy used by Paulesu et al. (2001)—would not identify this region with an orthography-specific deficit. In contrast, a comparison of the underactivation pattern (i.e., non-impaired > dyslexic) between Italian versus English would identify this region. The latter strategy is all the more sensible, given that there are known brain activation differences between Italian and English non-impaired readers (Paulesu et al., 2000), and dyslexia-related dysfunctions can be supposed to be associated with these very same regions.

Moreover, the strategy to search for orthography-specific differences in dyslexic under- or overactivation would yield reasonable results for the hypothetical situations illustrated in Figures 1B–D. Situation D deserves closer attention. Here, both non-impaired and dyslexic readers show a language-related effect, that is, higher reading-related activation in a shallow compared with a deep orthography. Crucially, however, there is no effect of dyslexia within a language. Therefore, this activation pattern should not be considered as showing an orthography-specific dyslexic abnormality pattern. Again, the proposed search strategy would yield a reasonable result because it would not identify a region with this activation pattern. With the knowledge from their previous study, that is, an orthography-specific effect in non-impaired readers (Paulesu et al., 2000), and the strategy to directly compare dyslexic readers across languages, it seems like Paulesu et al. (2001) searched for such a pattern of general language-related differences. This is, however, rather uninformative when it comes to orthography-specific dyslexic activation abnormality patterns.

A further possible explanation for why the Paulesu et al. (2001) and Silani et al. (2005) studies did not find orthography-specific dyslexic abnormalities relates to the small number of participants (six participants per group, per language, and per activation task) resulting in low statistical power (Button et al., 2013). In addition, as already suggested by Paulesu et al. (2001), the dyslexic readers may have used idiosyncratic and inter-individually heterogeneous reading strategies resulting in less consistent group-level brain activation. This would be in line with evidence for the engagement of inter-individually diverse neuronal networks for reading (Seghier et al., 2008; Kherif et al., 2009; Richardson et al., 2011). Finally, PET imaging (Paulesu et al., 2001) and the VBM method (Silani et al., 2005) are subject to inherent limitations such as low temporal and spatial resolution and reliance on block-designs (PET), and mis-registration of images, mis-classification of tissue, and a neuroanatomically unspecific measurement of local gray matter volume or density (e.g., Mechelli et al., 2005; Richlan et al., 2013b), which may obscure subtle and fine-grained orthography-specific differences.

## Predictions derived from functional neuroanatomical models

As argued above, it is not surprising that Paulesu et al. (2001) and Silani et al. (2005) did not find evidence for differences in the neurocognitive deficits between dyslexic readers in deep and shallow orthographies despite the documented orthography-specific effects in non-impaired readers (e.g., Paulesu et al., 2000; Das et al., 2011). As evident from the review of behavioral studies, the usual finding is that successful reading acquisition is based on the same cognitive components in deep and shallow orthographies. The general pattern of early contribution of PA and later contribution of RAN is independent of OD. What varies across languages is the degree to which these components contribute over time (Vaessen et al., 2010). In addition, it makes a difference whether reading accuracy or reading speed is assessed (Moll et al., 2014).

Functional neuroanatomical models of developmental dyslexia (e.g., Pugh et al., 2000; Richlan, 2012) provide a basis for testable hypotheses about different brain activation patterns in non-impaired and dyslexic readers between languages differing in OD. Although these models are not explicit in stating orthography-specific predictions, their architecture (e.g., which brain regions are engaged by certain cognitive processes) allows one to derive hypotheses about expected brain activation patterns. These model-based predictions will be described below. In line with the behavioral findings on the contribution of cognitive components to reading, activation of brain regions across orthographies is not a matter of all or none but rather a matter of degree. In addition, due to the usual spatial smoothness of functional brain imaging data, a higher level of brain activation can be expressed in larger spatial extent of activation clusters. Thus, the functional neuroanatomical models do not predict involvement of completely different brain regions across orthographies, but rather activation of the same brain regions to a different degree and extent (Pugh et al., 2005).

In addition to tuning of local brain activation it was put forward that the development of skilled reading relies on systems-level plasticity (i.e., on changes in the interactions between brain regions) (Schlaggar and McCandliss, 2007). The idea is that brain regions that are already partially active at the beginning of learning to read become better connected over time (both structurally and functionally), thus providing the basis for the development of skilled reading. This interactive specialization concept is incorporated in the predictions of the newer functional neuroanatomical model (Richlan, 2012). It relies on the many neuroimaging studies from the last years that investigated reading-related structural connectivity by means of diffusion tensor imaging (DTI; e.g., Ben-Shachar et al., 2007; Hoeft et al., 2011; Vandermosten et al., 2012; Boets et al., 2013; Thiebaut de Schotten et al., 2014) or functional and effective connectivity by means of both task-based and resting-state fMRI (e.g., Richardson et al., 2011; van der Mark et al., 2011; Koyama et al., 2013; Vogel et al., 2014; Schurz et al., 2014). In addition to this MRI-based research, valuable information on inter-regional functional coupling can be gained from the time course of activation of relevant brain regions via temporally precise techniques such as electroencephalography (EEG) and magnetoencephalography (MEG) (for a recent review see Carreiras et al., 2014).

## The classical model

**Figure 2:**
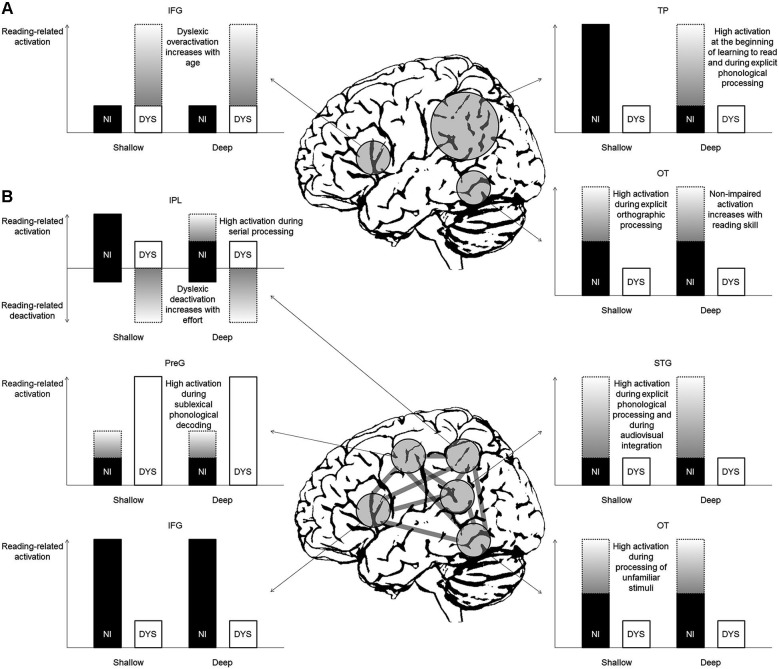
**Predictions for reading-related activation in non-impaired (NI) and dyslexic (DYS) readers in deep and shallow orthographies (A) based on the classical model by Pugh et al. (2000) and (B) based on the new model by Richlan (2012)**. Gray gradient bars represent activation that is only present under certain conditions (for detailed explanations see text). IFG = inferior frontal gyrus, IPL = inferior parietal lobule, OT = occipito-temporal cortex, PreG = precentral gyrus, STG = superior temporal gyrus, TP = temporo-parietal cortex.

Figure 2A illustrates the predictions for reading-related brain activation in non-impaired and dyslexic readers of deep and shallow orthographies based on the classical model by Pugh et al. (2000). This seminal model and its subsequent variations (e.g., McCandliss and Noble, 2003; Démonet et al., 2004; Sandak et al., 2004; Pugh et al., 2005) propose engagement of the left dorsal temporo-parietal (TP) cortex (including the posterior STG and the supramarginal and angular gyri of the IPL) during phonology-based reading processes (i.e., grapheme-phoneme conversion, phonological assembly) in non-impaired readers and a corresponding dysfunction (reflected in absent or reduced activation) in dyslexic readers. Consequently, for shallow orthographies, one would predict reading-related activation in non-impaired children and adults and underactivation in dyslexic readers. For deep orthographies, in contrast, one would expect activation only in non-impaired children or in tasks requiring phonology-based reading or explicit phonological analysis. The dyslexic readers, due to their proposed primary phonological TP deficit, would exhibit underactivation. In sum, the left dorsal TP system dominates at the beginning of learning to read in typically developing children, irrespective of orthography. Dyslexic readers, however, fail to properly activate this system.

Furthermore, the classical model proposes engagement of the left ventral OT cortex (including posterior ITG and FFG) during memory-based orthographic word recognition. In skilled readers, this system becomes the critical support for fast and efficient reading. In dyslexic readers, a secondary deficit of the left ventral OT system is assumed to follow from a primary deficit in left dorsal TP regions. The predictions for shallow orthographies are intermediate activation in non-impaired readers irrespective of reading age (unless explicit orthographic tasks require high engagement of this region) and little activation in dyslexic readers. For deep orthographies the predictions are strong activation in non-impaired adults and advanced children (that is, as soon as orthographic representations are built up) and underactivation in dyslexics. Therefore, the universal reading speed deficit of dyslexic readers across languages is thought to be reflected in underactivation of the left ventral OT cortex (Pugh, 2006).

Finally, the classical model includes a third anterior reading circuit, which is located in the left IFG. Its function is assumed to include (among others) speech-gestural articulatory recoding of print. According to the model and regardless of orthography, dyslexic readers should exhibit overactivation of this region and of additional right hemisphere posterior regions compared with non-impaired readers in order to compensate for their dysfunction in the two left posterior regions. This overactivation, however, is not present at the beginning of learning to read, but increases with age (Shaywitz et al., 2002).

## The new model

### Left inferior parietal lobule

Figure 2B illustrates the predictions for reading-related activation based on a newer model by Richlan (2012). The involved regions are largely the same (with some subtle anatomical variations) but the assumed functions in non-impaired reading and the associated dysfunctions in dyslexic reading are crucially different. Importantly, the model by Richlan (2012) divides the left TP circuit of Pugh et al. (2000) into a more dorsal IPL part adjacent to the intra-parietal sulcus and a more ventral STG part around the posterior sylvian fissure. The former was proposed to be engaged by more general attentional mechanisms, which are not exclusively related to reading (Shaywitz and Shaywitz, 2008). In the left dorsal IPL, as evidenced by meta-analysis, the typical finding is increased task-negative activation in dyslexic compared with non-impaired children during reading or reading-related processes (Richlan et al., 2011). That is, non-impaired readers show weak (de)activation relative to a low-level visual baseline, whereas dyslexic readers exhibit marked deactivation relative to baseline. This task-induced interruption of baseline activation was interpreted as reflecting greater mental effort during reading in dyslexic readers.

Note, however, that the left dorsal IPL can also be activated by non-impaired readers depending on the task and stimulus requirements. In this case, the typical finding is reduced task-positive activation in dyslexic readers (e.g., Cao et al., 2006; van der Mark et al., 2009; Richlan et al., 2010; Wimmer et al., 2010) and disrupted functional connectivity between the left dorsal IPL and the left ventral OT cortex (Cao et al., 2008; van der Mark et al., 2011). It is possible that the left dorsal IPL is involved in shifting attention from letter to letter within a string (e.g., Behrmann et al., 2004; Wager et al., 2004; Rosazza et al., 2009; Cabeza et al., 2012) and thus subserves serial decoding. This function is needed during reading based on grapheme-phoneme conversion but is irrelevant during reading based on whole-word representations. Accordingly, the left dorsal IPL was consistently identified with higher activation in response to pseudoword reading compared with word reading in a meta-analysis of 36 neuroimaging studies of typical readers (Taylor et al., 2013).

Assuming a functional role of the left dorsal IPL subserving serial decoding in non-impaired readers and a dysfunction in dyslexic readers, the prediction would be reduced dyslexic task-positive activation in shallow orthographies (with reliance on rule-based grapheme-phoneme conversion) or when serial processing is emphasized by task or stimulus demands (e.g., Cohen et al., 2008; Rosazza et al., 2009). In contrast, in deep orthographies (with reliance on memory-based word recognition) or when visual-orthographic whole-word processing is predominant, one would expect little engagement of the left dorsal IPL in non-impaired readers. Dyslexic readers, however, would exhibit increased task-negative activation (i.e., deactivation) in response to greater mental effort during reading, reflecting an interruption of the baseline activation of the left dorsal IPL (Richlan et al., 2011). Note that the orthography-specific predictions would be the same based on psycholinguistic grain size theory (Ziegler and Goswami, 2005, 2006). That is, non-impaired readers in shallow orthographies should rely more on the serial attention shifting mechanism in the left dorsal IPL compared with non-impaired readers in deep orthographies, due to the smaller size of the orthographic units. In order to distinguish whether dyslexic underactivation relative to non-impaired readers stems from differences in task-positive or task-negative activation, it is indispensable for future fMRI studies to include rest blocks (in the case of block-design fMRI) or appropriate inter-stimulus intervals and null-events (in the case of event-related fMRI).

With respect to neuroanatomy, it is important to note that the left dorsal IPL clusters found in the meta-analyses of dyslexic brain activation abnormalities (Richlan et al., 2009, 2011) correspond more to the supramarginal gyrus than to the angular gyrus. Among the functions discussed above, the former is thought to be involved in phonological processes, whereas the latter is thought to be involved in semantic processes (e.g., Vigneau et al., 2006; Binder et al., 2009; Cabeza et al., 2012; Carter and Huettel, 2013). This classical subdivision, however, might be too coarse (Seghier and Price, 2012). Recent evidence from studies on cytoarchitectonics (Caspers et al., 2006, 2008), receptor architectonics (Caspers et al., 2013), structural connectivity (Mars et al., 2011, 2012), and functional connectivity (Yeo et al., 2011; Bzdok et al., 2013) speaks for much more fine-grained parcellation of the parietal cortex into multiple subdivisions.

### Left superior temporal gyrus

The second left TP region (left posterior STG adjacent to the sylvian fissure) is not assigned a key role in grapheme-phoneme conversion in the new model. This assumption stands in marked contrast to the classical model, in which this region is proposed to dominate at the beginning of learning to read. Consequently, in the new model it is not assumed that a primary deficit in the left STG leads to a secondary deficit in the left ventral OT cortex in dyslexic readers. Instead, the left perisylvian TP region seems to be involved when explicit fine-grained phonological analysis is required (e.g., Griffiths and Warren, 2002; Hickok et al., 2011; DeWitt and Rauschecker, 2012; Price, 2012) or when information from auditory linguistic inputs and visual linguistic inputs (i.e., speech sounds and letters) has to be integrated (e.g., van Atteveldt et al., 2004; Blau et al., 2010). Hence, activation of this region is predicted when the task involves unimodal auditory or bimodal audiovisual processing. For unimodal visual processing, engagement of this region is typically not reported, unless the task involves demanding phonological analysis. In a recent meta-analysis the left STG was not considered to be part of the reading network (Taylor et al., 2013).

There is emerging evidence that the neural correlates of multisensory letter-speech sound integration might be modulated by OD. Specifically, a study with English adult readers (Holloway et al., 2013) did not find congruency effects for letter-speech sound pairs in the STG as were previously reported in a similar study with Dutch adult readers (van Atteveldt et al., 2004). With respect to brain plasticity, it was shown that reading development has an influence on activation in the left STG regions associated with phonological processing, and that this influence is stronger in alphabetic compared with logographic writing systems (Brennan et al., 2013). In addition, a recent meta-analysis on structural brain abnormalities in dyslexia identified reduced gray matter volume in bilateral perisylvian TP regions, possibly reflecting reduced tuning of the phonological network as a result of reduced reading experience in dyslexics (Richlan et al., 2013b). Therefore, one may speculate that, opposite to the developmental assumption of the classical model, a primary left ventral OT dysfunction results in a secondary left STG dysfunction in dyslexia. The influence of OD within alphabetic writing systems on this developmental effect, however, is still a blank spot on the map.

### Left ventral occipito-temporal cortex

One may ask where in the brain, if not in the left perisylvian TP cortex, grapheme-phoneme conversion should be located in the new model. The idea is that the left ventral OT cortex is associated with both visual-orthographic whole-word processing and serial grapheme-phoneme conversion. Among others (e.g., Xu et al., 2001; Mechelli et al., 2003; Binder et al., 2005; Kronbichler et al., 2007, 2009; Bruno et al., 2008; Brem et al., 2010; Ludersdorfer et al., 2013), evidence comes from an fMRI study (Schurz et al., 2010), in which non-impaired German readers exhibited a length by lexicality interaction in the left ventral OT cortex (i.e., an increase of activation with increasing number of letters for pseudowords but not for words). German dyslexic readers exhibited overall lower activation and failed to show the modulation of activation by length of pseudowords (Richlan et al., 2010).

At least for German, other fMRI studies have also shown that dyslexic underactivation is more pronounced when orthographically unfamiliar stimuli (e.g., pseudowords or pseudohomophones) impose higher demands on phonological processing compared with orthographically familiar stimuli (words) (van der Mark et al., 2009; Wimmer et al., 2010). Future studies in other shallow orthographies (e.g., Dutch, Italian, or Spanish) are expected to yield similar results. Therefore, the prediction for shallow orthographies is intermediate activation for non-impaired readers with increasing activation when grapheme-phoneme conversion is required by task or stimulus demands (e.g., pseudowords). Dyslexic readers are expected to exhibit weak overall activation and failure to increase activation in response to unfamiliar stimuli.

Higher left ventral OT cortex activation for unfamiliar compared with familiar letter strings is a common finding also in the English-based literature and was interpreted as a reflection of sustained task-related top-down processing (Dehaene and Cohen, 2011). A different explanation was put forward by Price and Devlin (2011). In their interactive account, higher activation for unfamiliar compared with familiar letter strings in the left ventral OT cortex is explained by higher prediction error (i.e., the difference between bottom-up visual information and top-down predictions). The top-down predictions are generated automatically from prior experience in higher cortical levels that contribute to representing phonology, semantics, and actions. This view is in line with the role of the left ventral OT cortex in grapheme-phoneme conversion in the new model. In the Interactive Account, the left ventral OT underactivation exhibited by dyslexic readers is interpreted as failure to establish hierarchical connections and access top-down predictions. As top-down predictions from phonology and semantics play an important role in reading irrespective of OD, the left ventral OT activation pattern is expected to be similar in deep and shallow orthographies.

### Left inferior frontal gyrus

A further difference between the classical model (Pugh et al., 2000) and the new model (Richlan, 2012) refers to the left anterior reading component. In contrast to the classical model, the new model—supported by findings from meta-analyses (Richlan et al., 2009, 2011)—proposes a subdivision of the left anterior system into an IFG region and a dorsal precentral region. The former was consistently identified with dyslexic underactivation, whereas the latter was consistently identified with dyslexic overactivation.

The left IFG underactivation is thought to reflect the problem of dyslexic readers to access phonological output representations (Ramus and Szenkovits, 2008). This notion was recently supported by a study combining multivoxel pattern analysis, and functional and structural connectivity analysis (Boets et al., 2013). The main finding was reduced functional coupling in an auditory phoneme discrimination task and reduced white matter integrity as measured by DTI between left IFG and STG regions in dyslexic readers. In addition, the left IFG is assumed to have strong reciprocal connections and to interact with the left ventral OT cortex during non-impaired reading (e.g., Catani et al., 2005; Ben-Shachar et al., 2007; van der Mark et al., 2011; Vandermosten et al., 2012; Yeatman et al., 2013; Schurz et al., 2014).

Up to now, there are no indications of essential differences in dyslexic underactivation in shallow versus deep orthographies in the left IFG. Some accounts speak for engagement of the left IFG in grapheme-phoneme conversion (e.g., Jobard et al., 2003) or lexical access (e.g., Heim et al., 2013). There is, however, room for speculation because the IFG is a heterogeneous region, which is not only characterized by anatomical subdivisions based on neurotransmitter receptor architectonics (Amunts et al., 2010), but was associated with various different cognitive and emotional processes (e.g., Laird et al., 2011; Price, 2012; Richlan et al., 2013a).

### Left dorsal precentral gyrus

In line with the classical model, the left dorsal PreG was consistently identified with overactivation in dyslexic children and adults (Richlan et al., 2009, 2011). This overactivation is assumed to reflect compensatory reliance on articulatory processes during reading from an early age on. The left dorsal PreG is part of the sublexical phonological decoding route and typically identified with higher activation for pseudowords compared with words in non-impaired readers (Price, 2012; Taylor et al., 2013). Interestingly, in our study with dyslexic adolescents and young adults (Richlan et al., 2010), it was the only region which showed higher activation for pseudowords compared with words together with a length effect for pseudowords in dyslexic readers. With respect to OD, no differences are assumed in left dorsal PreG overactivation between dyslexic readers in deep and shallow orthographies, because of universal overreliance on articulatory processes.

### Functional integration/interactive specialization

Following Schlaggar and McCandliss (2007), the new model incorporates the concept of interactive specialization, that is, the idea that the development of skilled reading relies on the functional integration of distributed brain regions. Changes through development are not only assumed to take place in local brain modules (reflected in tuning of regional activation patterns and structural cortical plasticity), but also on the systems-level (reflected in alterations in functional coupling and white matter connectivity between brain regions). As already mentioned in Section Predictions Derived from Functional Neuroanatomical Models, a number of studies investigated the functional and structural neuroanatomy of reading from this systems-level perspective.

A main focus was on connectivity of the left ventral OT cortex with other language-related brain regions. There is good evidence from studies with non-impaired readers (e.g., Koyama et al., 2011; Vogel et al., 2012), developmental dyslexic readers (e.g., Shaywitz et al., 2003; van der Mark et al., 2011), and acquired dyslexic readers (e.g., Epelbaum et al., 2008; Seghier et al., 2012; Woodhead et al., 2013), that integration of the left ventral OT cortex with frontal and parietal regions is vital for fast and efficient reading (Price and Devlin, 2011). In addition to functional integration, it was shown that skilled adult readers show functional segregation (i.e., decoupling) of the reading network with the typically task-negative default mode network (Koyama et al., 2011).

The connections between brain regions in Figure 2B should be taken as illustration of the interactive specialization framework. For reasons of simplicity, all possible connections between brain regions are drawn, but the assumption is not that all of the brain regions interact with each other in any given situation. Instead, the idea is that different parts of the overall network interact in flexible and temporal dynamic ways depending on the required cognitive processes for a given task or stimulus.

Based on the evidence available up to now, it is impossible to reliably predict differences in connectivity patterns between deep and shallow orthographies. Studies aimed at these differences, however, are likely to shed light on the functional neuroanatomical reflection of OD, despite potentially very subtle differences in local brain activation profiles. Therefore, I look forward to innovative future studies investigating the effect of OD within alphabetic writing systems and differences between alphabetic and other writing systems by means of structural, functional, and effective connectivity in task-based as well as resting-state fMRI.

## Summary and conclusion

To sum up, dyslexia-related differences between deep and shallow orthographies can be expected in a variety of left hemisphere brain regions, depending on task and stimulus demands and age of participants. The two models (Pugh et al., 2000; Richlan, 2012) differ in many respects as for how they predict the degree and extent of engagement in these regions. In addition, differences between deep and shallow orthographies are likely to be reflected in the dynamic interactions between brain regions.

Evidence from cross-linguistic brain imaging studies on developmental dyslexia is scarce. The different approaches of classical between-subjects designs, within-subjects designs (in the case of bilingual participants), and artificial orthography learning paradigms should be continued and expanded in the future. In addition, meta-analysis might provide a valuable tool to synthesize and compare a high number of original studies, which were conducted within a single language. A comparable strategy was already successfully applied in the investigation of child and adult studies of developmental dyslexia (Richlan et al., 2011).

The investigation of typical and atypical reading processes in different orthographies yields important implications for the neurobiological understanding of developmental dyslexia. The given variations in OD and the role of English as an “outlier” orthography (Share, 2008) should be considered as an opportunity to test the current neurocognitive models and to refine them. The present review article contributes to this endeavor by providing orthography-specific predictions derived from two distinct conceptions of the functional neuroanatomy of non-impaired and dyslexic reading. These predictions should be tested in future brain imaging studies of reading.

## Conflict of interest statement

The author declares that the research was conducted in the absence of any commercial or financial relationships that could be construed as a potential conflict of interest.
